# Divalent ligand-monovalent molecule binding

**DOI:** 10.1039/d1sm00070e

**Published:** 2021-04-26

**Authors:** Mathijs Janssen, Harald Stenmark, Andreas Carlson

**Affiliations:** Department of Mathematics, Mechanics Division, University of Oslo N-0851 Oslo Norway mathijsj@uio.no acarlson@math.uio.no; Centre for Cancer Cell Reprogramming, Faculty of Medicine, University of Oslo Montebello N-0379 Oslo Norway

## Abstract

Simultaneous binding of a divalent ligand to two identical monovalent molecules is a widespread phenomenon in biology and chemistry. Here, we describe how two such monovalent molecules B bind to a divalent ligand AA to form the intermediate and final complexes AA·B and AA·B_2_. Cases wherein the total concentration [AA]_*T*_ is either much larger or much smaller than the total concentration [B]_*T*_ have been studied earlier, but a systematic description of comparable concentrations [AA]_*T*_ and [B]_*T*_ is missing. Here, we present numerical and analytical results for the concentrations [AA·B] and [AA·B_2_] for the entire range 0 < [B]_*T*_/[AA]_*T*_ < ∞. Specifically, we theoretically study three types of experimental procedures: dilution of AA and B at fixed [B]_*T*_/[AA]_*T*_, addition of AA at fixed [B]_*T*_, and addition of B at fixed [AA]_*T*_. When [AA]_*T*_ and [B]_*T*_ are comparable, the concentrations of free ligands and molecules both decrease upon binding. Such depletion is expected to be important in cellular contexts, *e.g.*, in antigen detection and in coincidence detection of proteins or lipids.

## Abstract 2

Simultaneous binding of a divalent ligand to two identical monovalent molecules is a widespread phenomenon in biology and chemistry. This article presents results for the concentrations of bound complexes for arbitrary ratios of ligand-to-molecule concentrations.

## Graphical abstract

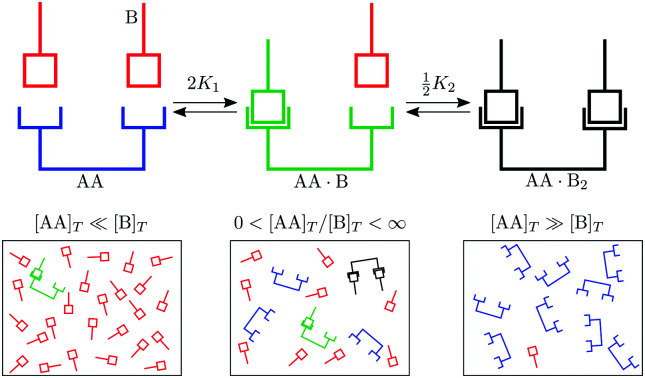

## 1 Introduction

Chemical binding is at the heart of many processes in biology, including oxygen binding to haemoglobin, self assembly, antibodies binding to antigens, and growth factors binding to their transmembrane receptors.^1–7^ In many cases, binding interactions should be specific and strong, yet reversible.^8–11^ One way to accomplish such a “molecular velcro” is through ligands containing many ligating units per molecule: Multivalent ligands are known to bind some transmembrane receptors more readily than their monovalent counterparts (with one binding site per ligand).^8^ This makes multivalent ligands interesting in clinical applications, where less therapeutic cargo is needed for the same response. The intuitive explanation why multivalent ligands can bind more readily to some receptors on a plasma membrane or a viral envelope goes as follows. After the binding of a first ligating unit with association constant *K*_1_, other ligating units of a multivalent ligand are close to other membrane-bound receptors as well. Around a first bound unit, a second ligating unit is thought to sweep out a semi circle with a radius set by the (fixed) distance between ligating units.^12–15^ As this distance is a few nanometers at most, the *effective concentration* of ligating units belonging to a partly-bound multivalent ligand is much higher than the concentration of unbound ligands nearby. More generally, for flexible rather than stiff linkers between ligating units,^16,17^ effective concentrations can be determined rigorously within statistical mechanics.^18,19^

In turn, high effective concentrations are reflected in a high association constant *K*_2_ for binding a second ligating unit of a multivalent ligand, and the same for further binding steps. Systems for which *K*_2_/*K*_1_ > 1 are called *cooperative*.^20–23^ In the above example of large effective concentrations, one speaks of apparent cooperativity. This is to distinguish it from true cooperativity based on allostery,^24^ which refers to binding pockets whose binding affinity changes when nearby pockets are occupied, as happens for the binding of oxygen to haemoglobin.^25^ In either way, the hallmark of cooperativity is the switching from mostly-unbound to mostly-bound ligands over a narrow concentration range.^20^

Equations for the concentrations of molecules involved in binding reactions are typically nonlinear and with a high polynomial order. In two simple cases—the binding of a monovalent ligand to a monovalent receptor^1,3^ and the binding of two different monovalent ligands to one type of monovalent protein^26^—the concentrations of all involved species can be expressed analytically nonetheless. For more complicated reactions, analytical progress is often only possible if one molecular species is assumed to be in excess as compared to other species.^20,27^ This limit is only appropriate to certain systems and experiments. If no molecular species is in excess as compared to the other present in the system, the full reaction-rate equations should be solved, and binding will deplete the unbound species.

**Fig. 1:**
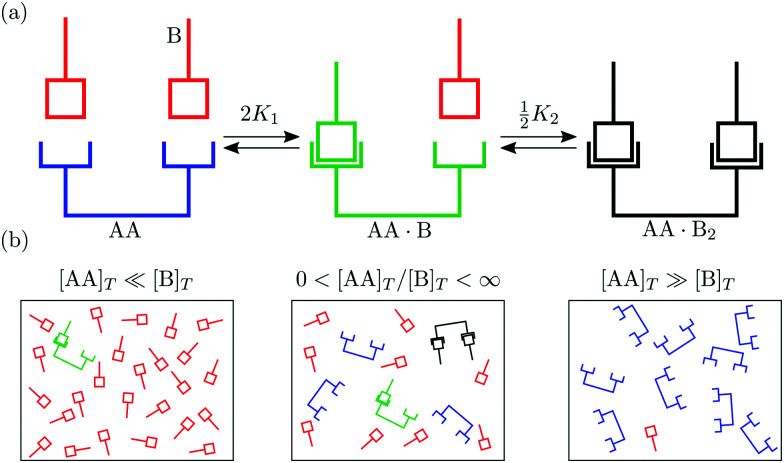
(a) Binding of two monovalent molecules B to a divalent ligand AA, to form the complexes AA·B and AA·B_2_. (b) Different relative concentrations of [AA]_*T*_ and [B]_*T*_.

In this article we explore the interplay between multivalency, cooperativity, and depletion. We do so by discussing the reversible binding of a divalent ligand AA to two identical monovalent molecules B [Fig. 1(a)],

1 AA + 2B ⇌ AA·B + B ⇌ AA·B_2_,

as it is the simplest binding reaction that can display nontrivial effects of multivalency and cooperativity.^20,21,23^Eqn (1) also has value in its own right: It captures hormone action,^28^ the binding of divalent antibodies to antigens on pathogens,^12,13,27,29–32^ and it was realised in synthetic systems.^16^ In many biological systems to which eqn (1) may be relevant, B may represent a protein or a cell membrane receptor. Yet, to keep our discussion completely general, we refer to B simply by “molecule”. We denote the total volumetric concentration of ligands AA and molecules B⁠—both bound and unbound—by [AA]_*T*_ and [B]_*T*_. Most prior works studied the reaction in eqn (1) assuming either [AA]_*T*_ ≪ [B]_*T*_ or [AA]_*T*_ ≫ [B]_*T*_ [Fig. 1(b)]. For instance, Hunter and Anderson^20^ asserted that the concentration of monovalent molecules is hardly affected ([B] ≈ [B]_*T*_) by the reaction in eqn (1) if it happens at [AA]_*T*_ ≪ [B]_*T*_; Perelson and DeLisi^27^ asserted that the concentration of divalent ligands is hardly affected ([AA] ≈ [AA]_*T*_) by the reaction in eqn (1) if it happens at [AA]_*T*_ ≫ [B]_*T*_. As we move away from these limits, neither [AA] ≈ [AA]_*T*_ nor [B] ≈ [B]_*T*_ can hold as the reaction in eqn (1) will deplete both the free ligands AA and the molecules B. Here, we study the binding of divalent ligands AA to monovalent molecules B over the complete range 0 < [B]_*T*_/[AA]_*T*_ < ∞.

## 2 Model

The reaction in eqn (1) does not affect the total number of AA and B molecules, which gives the following particle-conservation constraints

2a [AA]_*T*_ = [AA] + [AA·B] + [AA·B_2_],

2b [B]_*T*_ = [B] + [AA·B] + 2[AA·B_2_].

In Appendix A we show how the reaction-rate equations associated with eqn (1) reduce at steady state to3
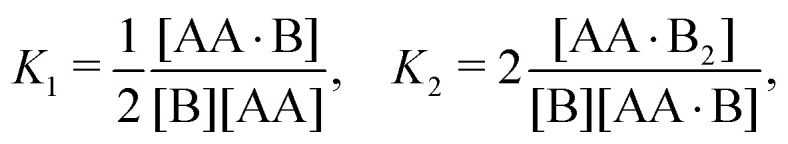
where *K*_1_ and *K*_2_ are the association constants, and where factors of 1/2 and 2 account for the degeneracy of the intermediate complex AA·B. While ref. 13, 20, 21 and 30 used the same convention, ref. 27 absorbed the factor 1/2 into *K*_1_, and ref. 29 and 32 absorbed the factors 1/2 and 2 into *K*_1_ and *K*_2_.

To model divalent antibody binding to monovalent surface-bound antigens, ref. 12 and 13 expressed concentrations of antigens and (partly) bound complexes in numbers per unit area.^13,29^ Yet, the governing equations of ref. 13 and 29 could also be cast into the form of eqn (2) and (3), that is, with volumetric concentrations *only*, and the effect of reduced positional freedom of surface-bound molecules absorbed into the constants *K*_1_ and *K*_2_. Hence, though volumetric concentrations appear in our eqn (2) and (3), this set of equations can just as well describe a binding process wherein either AA or B is confined to a thin (membrane) surface (see also page 13 of ref. 1). Still, an assumption underlying the derivation of eqn (3) in terms of concentrations is that all species are well mixed. This assumption may be violated for certain types of B molecules, for instance, receptors that cluster at the plasma membrane.^33,34^

From the four expressions in eqn (2) and (3) we can determine the four unknown concentrations [AA], [B], [AA·B], and [AA·B_2_] in terms of the four physical parameters *K*_1_, *K*_2_, [AA]_*T*_, and [B]_*T*_. First, we eliminate [AA] and [B] from eqn (3) with eqn (2),

4a [AA·B] = 2*K*_1_([B]_*T*_ − [AA·B] − 2[AA·B_2_]) × ([AA]_*T*_ − [AA·B] − [AA·B_2_]),

4b
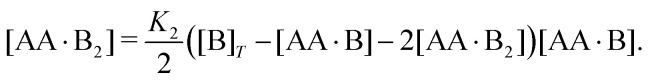
Next, we rewrite eqn (4b) to5
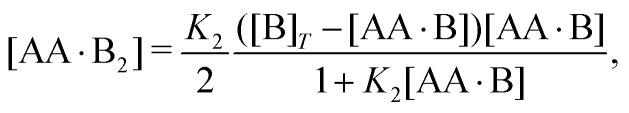
with which we eliminate [AA·B_2_] from eqn (4a),*a*[AA·B]^3^ + *b*[AA·B]^2^ + *c*[AA·B] + *d* = 0,*a* ≡ *K*_2_(*K*_1_ − *K*_2_),*b* ≡ 2(*K*_1_ − *K*_2_ − [AA]_*T*_*K*_1_*K*_2_),*c* ≡ 2*K*_1_[AA]_*T*_(*K*_2_[B]_*T*_ − 1) − 2*K*_1_[B]_*T*_ − *K*_1_*K*_2_[B]_*T*_^2^− 1,

6 *d* ≡ 2*K*_1_[AA]_*T*_[B]_*T*_.

The cubic eqn (6) for [AA·B] can be solved analytically with Cardano's formula. Unfortunately, its solution for general *K*_1_, *K*_2_, [AA]_*T*_, and [B]_*T*_, presented in Appendix B, is too cumbersome to be of use. We therefore also present analytical solutions to eqn (5) and (6) for specific (limiting) values of *K*_1_, *K*_2_, [AA]_*T*_, and [B]_*T*_ in Appendices C–E. First, Appendix C covers the case *K*_2_ = *K*_1_. The cubic term in eqn (6) then vanishes, leaving behind a quadratic equation that can be easily solved analytically for [AA·B] [see eqn (C2a)]. Also [AA·B_2_] and the “occupancy”^20^*θ* ≡ ([AA·B]/2 + [AA·B_2_])/[AA]_*T*_ are governed by simple expressions [see eqn (C4)]. Intuitively, for *K*_2_ = *K*_1_, each divalent ligand AA acts as two independent monovalent ligands A binding to two molecules B: *θ* coincides with a literature expression^3^ for the concentration of bound A·B at a molecule-to-ligand ratio [B]_*T*_/(2[A]_*T*_). Second, Appendix D covers the case [AA]_*T*_ ≪ [B]_*T*_. We rederive Hunter and Anderson's results^20^ for [AA·B] and [AA·B_2_] and show that they contain errors of order 𝒪([AA]_*T*_/[B]_*T*_). Last, Appendix E covers the case [AA]_*T*_ ≫ [B]_*T*_. For this case, we solve eqn (6) with a power series approximation to [AA·B]. Our solutions to [AA·B] and [AA·B_2_] differ from Perelson and DeLisi's results^27^ from 𝒪([B]_*T*_^3^/[AA]_*T*_^3^) onwards.

While in eqn (6) we isolated [AA·B] from eqn (2) and (3), we could have also chosen to isolate [B] instead. Indeed, cubic equations for [B] were reported in eqn (S) of ref. 13 and eqn (25) of ref. 29 [which we rederive in Appendix F]. However, neither of those articles discussed the dependence of [AA·B] and [AA·B_2_] on *K*_1_, *K*_2_, [AA]_*T*_, and [B]_*T*_ in as much detail as we do below.

## 3 Results

We present numerical results for [AA·B] and [AA·B_2_] from eqn (5) and (6) for different choices of fixed and varied *K*_1_, *K*_2_, [AA]_*T*_, and [B]_*T*_. Specifically, we mimic a dilution experiment, wherein we vary [AA]_*T*_ and [B]_*T*_ at fixed [B]_*T*_/[AA]_*T*_; a titration-like experiment, wherein we vary [AA]_*T*_ at fixed [B]_*T*_; and another titration-like experiment, wherein we vary [B]_*T*_ at fixed [AA]_*T*_. While the concentrations [AA]_*T*_ and [B]_*T*_ can be experimentally varied over decades, *K*_1_ and *K*_2_ are set by fixed molecular properties.^18,19^ Accordingly, we mostly consider different but fixed values of the “cooperativity parameter” *α* = *K*_2_/*K*_1_. *α* is related to the free energy of interaction between sites, see eqn (10) of ref. 20. We reinforce our numerical solutions of eqn (5) and (6) by the aforementioned analytical expressions for specific parameter values [see Appendices C–E].

### 3.1 Diluting a solution of AA and B at fixed [B]_*T*_/[AA]_*T*_

**Fig. 2:**
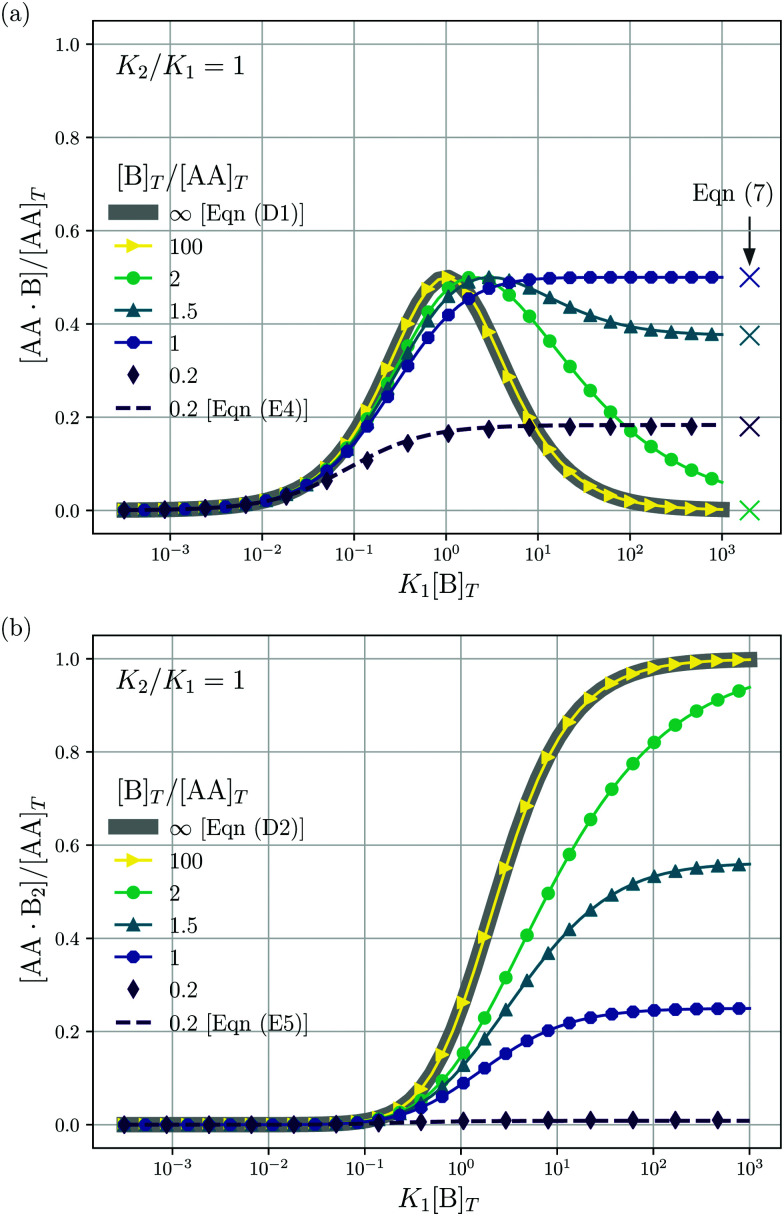
Theoretical predictions for a dilution experiment, wherein [AA]_*T*_ and [B]_*T*_ vary at fixed [B]_*T*_/[AA]_*T*_, *K*_1_, and *K*_2_. We show [AA·B]/[AA]_*T*_ (a), [AA·B_2_]/[AA]_*T*_ (b) as a function of *K*_1_[B]_*T*_ for *K*_2_/*K*_1_ = 1 and [B]_*T*_/[AA]_*T*_ = 0.2,1,1.5,2.0 and 100. Also shown are approximations to [AA·B]/[AA]_*T*_ and [AA·B_2_]/[AA]_*T*_ for [B]_*T*_ ≫ [AA]_*T*_ [eqn (D1) and (D2)] and for [B]_*T*_ ≪ [AA]_*T*_ [eqn (E4) and (E5)]. Panel (a) shows the analytical predictions from eqn (7) for *K*_1_[B]_*T*_ ≫ 1 with crosses.

We consider a solution with initial concentrations [AA]_*T*_ and [B]_*T*_ to which solvent is added. In such a dilution experiment, [AA]_*T*_ and [B]_*T*_ decrease at fixed [B]_*T*_/[AA]_*T*_, *K*_1_, and *K*_2_. Fig. 2 shows numerical results for [AA·B]/[AA]_*T*_ (a) and [AA·B_2_]/[AA]_*T*_ (b) as a function of *K*_1_[B]_*T*_, for several [B]_*T*_/[AA]_*T*_ and *K*_2_ = *K*_1_. First, we see that the numerical solutions for [B]_*T*_/[AA]_*T*_ = 100 (yellow triangles and lines) are close to Hunter and Anderson's predictions [eqn (D1) and (D2)], indicated by thick grey solid lines. For [B]_*T*_/[AA]_*T*_ = 100 and *K*_1_[B]_*T*_ = 10^3^, we evaluated that [B] = 0.98[B]_*T*_; hence, the assumption [B] = [B]_*T*_ of ref. 20 is satisfied to a high degree at this [B]_*T*_/[AA]_*T*_ value. Second, the numerical results for [B]_*T*_/[AA]_*T*_ = 0.2 (purple diamonds) are close to Perelson and DeLisi's predictions eqn (E4) and (E5) (purple dashed lines). Yet, we observe tiny differences between the numerical predictions and eqn (E4) around *K*_1_[B]_*T*_ = 1 in panel (a). This observation reinforces our analytical insight of Appendix E, namely, that eqn (E4) and (E5) contain errors of 𝒪([B]_*T*_^3^/[AA]_*T*_^3^). For [B]_*T*_/[AA]_*T*_ = 0.2 and *K*_1_[B]_*T*_ = 10^3^, we evaluated that [AA] = 0.81[AA]_*T*_; hence, the assumption [AA] = [AA]_*T*_ of ref. 27 is satisfied to some extend at this [B]_*T*_/[AA]_*T*_ value. Comparing to our earlier evaluation of [B] at [B]_*T*_/[AA]_*T*_ = 100, we see that, as anticipated in the introduction, the closer [B]_*T*_/[AA]_*T*_ is to unity, the stronger the unbound species are depleted. Third, a salient feature of the curves in Fig. 2(a) are the plateaus for *K*_1_[B]_*T*_ ≫ 1 and [B]_*T*_ ≈ [AA]_*T*_. As we derive in Appendix C [specifically, eqn (C3)], their height is set by7
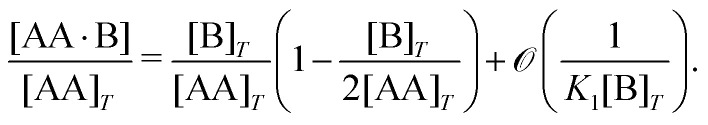
We indicate the predictions from eqn (7) with crosses in Fig. 2(a). The plateau height in Fig. 2(a) is maximal for [B]_*T*_ = [AA]_*T*_, as also follows from eqn (7). Fourth, note that [AA·B] cannot exceed the total concentrations of its constituents [AA]_*T*_ and [B]_*T*_; hence, 0 < [AA·B]/[AA]_*T*_ < min(1,[B]_*T*_/[AA]_*T*_). Likewise, for [AA·B_2_], we find that 0 < [AA·B_2_]/[AA]_*T*_ < min(1,[B]_*T*_/(2[AA]_*T*_)). The data in Fig. 2 satisfies these constraints.

**Fig. 3:**
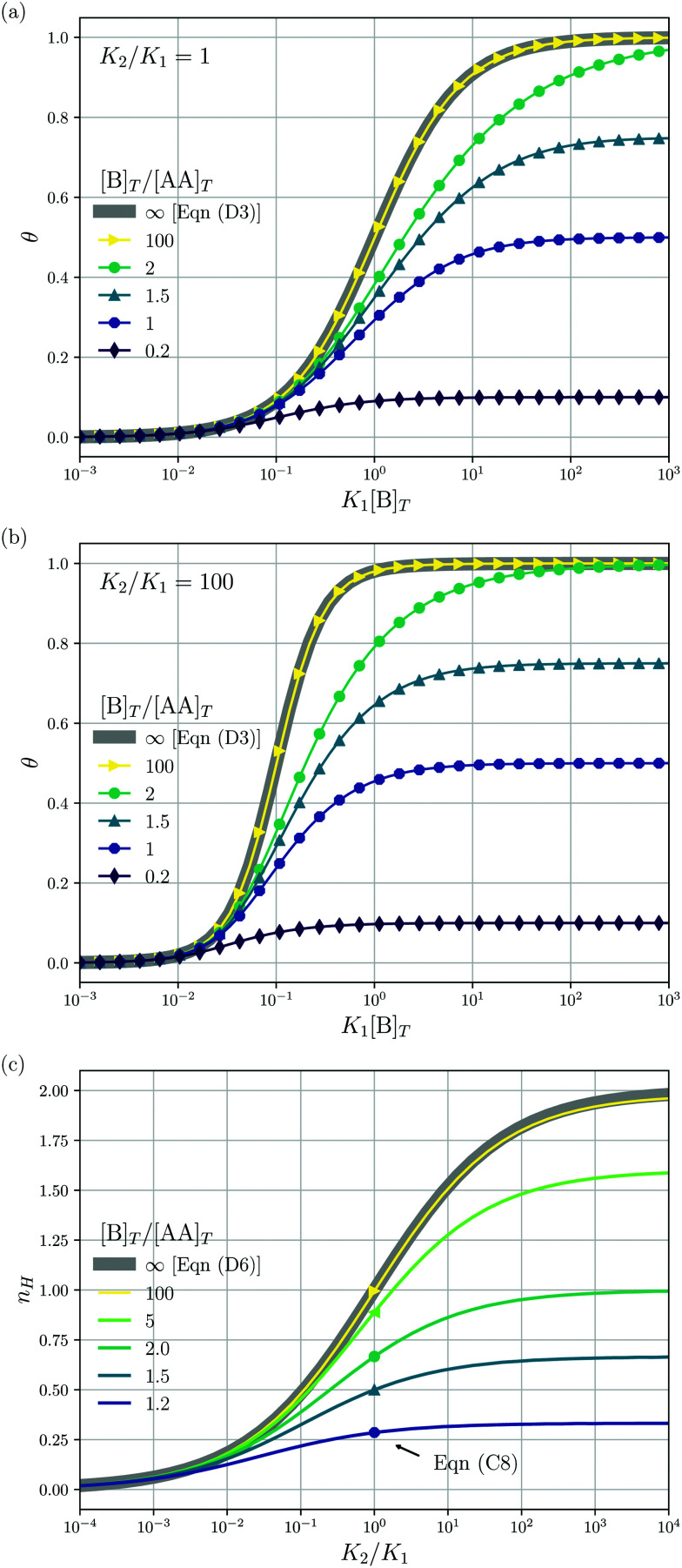
The occupancy *θ* for *K*_2_/*K*_1_ = 1 (a) and *K*_2_/*K*_1_ = 100 (b) and other parameters as in Fig. 2. Panel (c) shows the Hill coefficient *n*_H_ [eqn (8)] for several [B]_*T*_/[AA]_*T*_ > 1 (lines) and predictions for *K*_2_ = *K*_1_ of the analytical expression eqn (C8) (symbols). The thick grey lines represent eqn (D3) [(a and b)] and eqn (D4) (c), corresponding to [B]_*T*_/[AA]_*T*_ → ∞.

**Fig. 4:**
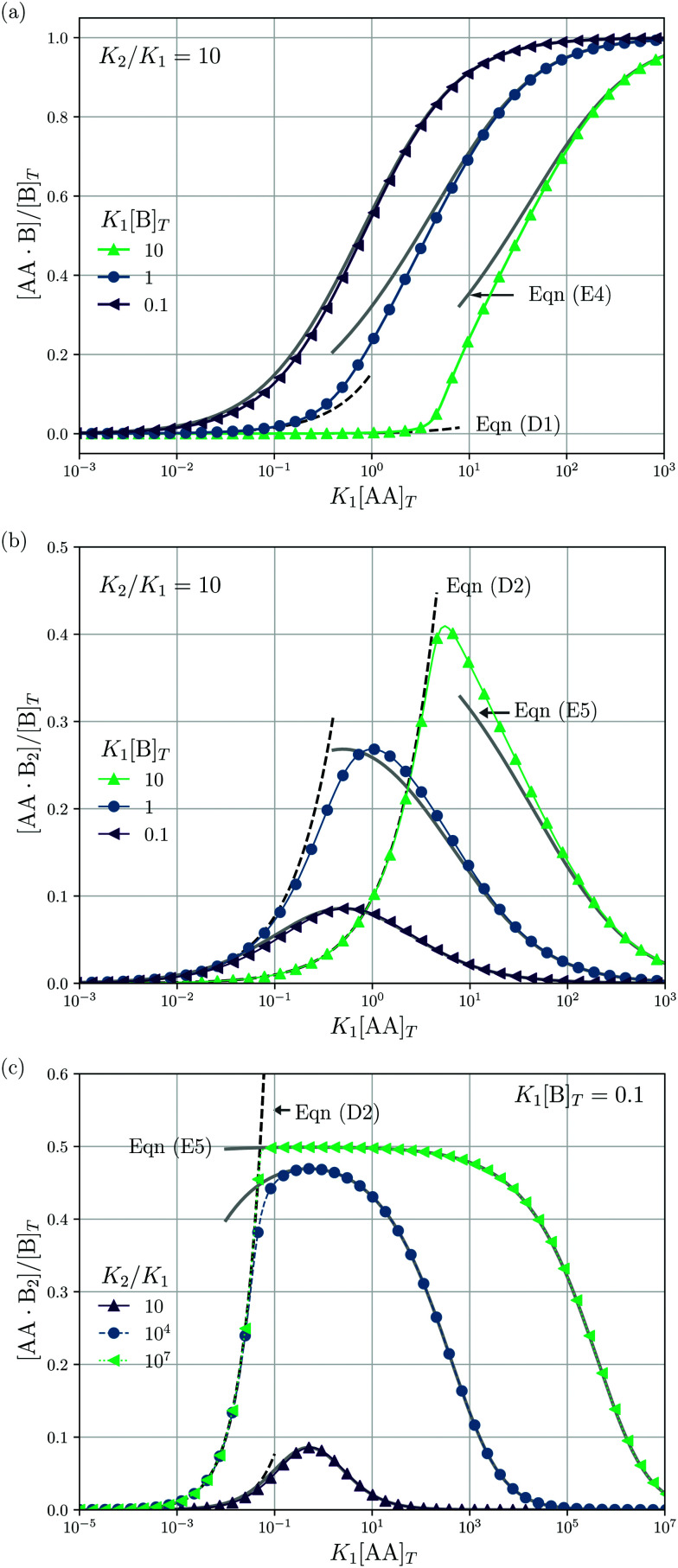
Theoretical predictions for a titration-like experiment wherein [AA]_*T*_ increases at fixed *K*_1_, *K*_2_, and [B]_*T*_. We show [AA·B]/[B]_*T*_ (a) and [AA·B_2_]/[B]_*T*_ [(b and c)] as a function of *K*_1_[AA]_*T*_ for *K*_2_/*K*_1_ = 10 and several *K*_1_[B]_*T*_ [(a and b)] and for *K*_1_[B]_*T*_ = 0.1 and several *K*_2_/*K*_1_ (c). Panel (a) also shows eqn (E4) (grey lines) and eqn (D1) (grey dashed lines); panels (b and c) also show eqn (E5) (grey solid lines) and eqn (D2) (black dashed lines).

Fig. 3 shows the occupancy *θ* for *K*_2_/*K*_1_ = 1 (a) and *K*_2_/*K*_1_ = 100 (b) and other parameters as in Fig. 2. For [B]_*T*_/[AA]_*T*_ = 100, we again observe good agreement between Hunter and Anderson's expression [eqn (D3)] and the numerical data for *θ*, both for *K*_2_/*K*_1_ = 1 and *K*_2_/*K*_1_ = 100. Next, we see that increasing the cooperativity parameter *K*_2_/*K*_1_ shifts *θ* curves to smaller *K*_1_[B]_*T*_ values and that *θ* switches from *θ* ≈ 0 to *θ* ≈ 1 over a narrower *K*_1_[B]_*T*_ range⁠—the hallmark of cooperativity. To characterise the slope of *θ*, we numerically determined the Hill coefficient8
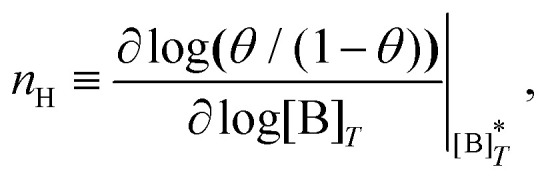
where 
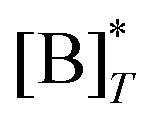
 is the molecular concentration at half occupancy, 
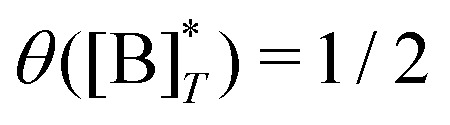
. Fig. 3(c) shows the *K*_2_/*K*_1_ dependence of *n*_H_ for several [B]_*T*_/[AA]_*T*_. As such, Fig. 3(c) generalises Fig. 6 of ref. 20, where [B]_*T*_ ≫ [AA]_*T*_ was considered. In that case, *n*_H_ is given by 
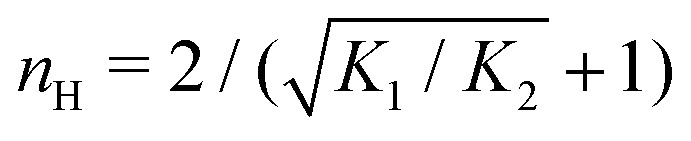
 [*cf.*eqn (D4)], indicated in Fig. 3(c) with a thick grey solid line. We see that, for [B]_*T*_/[AA]_*T*_ = 100, the numerically determined *n*_H_ is close to predictions from eqn (D4). Conversely, we see that *n*_H_ → 0 for [B]_*T*_/[AA]_*T*_ → 1. Fig. 4(a) and (b) show that half occupancy (*θ* = 1/2) is not reached if [B]_*T*_/[AA]_*T*_ < 1; hence, *n*_H_ is undetermined for [B]_*T*_/[AA]_*T*_ < 1. The symbols in Fig. 3(c) for *K*_2_ = *K*_1_ represent the analytical expression eqn (C8). These symbols match perfectly to the numerical *n*_H_ predictions.

### 3.2 Adding AA to a solution of B

Next, we study a case wherein [AA]_*T*_ is varied at fixed [B]_*T*_, *K*_1_, and *K*_2_. These conditions hold approximately in a titration experiment wherein a concentrated solution of AA is added to a dilute solution of B—provided that the “titrant” AA barely affects the volume of the B-containing solution; hence, neither affects [B]_*T*_. While eqn (6) could just as well be solved for a case wherein [B]_*T*_ decreases as [AA]_*T*_ increases, for clarity, we prefer to keep [B]_*T*_ fixed here. Clearly, starting from [AA]_*T*_ = 0 and adding much AA, we can span the complete range of 0 < [B]_*T*_/[AA]_*T*_ < ∞. The expressions derived in Appendices D and E for [B]_*T*_ ≫ [AA]_*T*_ and [B]_*T*_ ≪ [AA]_*T*_ can thus be expected to hold either at the start or the end of this titration-like experiment. Fig. 4 shows numerical results for [AA·B]/[B]_*T*_ (a) and [AA·B_2_]/[B]_*T*_ [(b) and (c)] as a function of *K*_1_[AA]_*T*_ for *K*_2_/*K*_1_ = 10 and several *K*_1_[B]_*T*_ [(a) and (b)] and for *K*_1_[B]_*T*_ = 0.1 and several *K*_2_/*K*_1_ (c). These panels display a well-documented effect: given a limited amount of B molecules, saturating a solution with AA will make the doubly-bound complex AA·B_2_ rare compared to its singly-bound counterpart AA·B, which is reflected in bell-shaped [AA·B_2_]/[B]_*T*_ curves.^1,27^ Intuitively, when the B molecules have many AA ligands to choose from, it is unlikely that two Bs will bind the same divalent ligand. However, for a very large cooperativity parameter *K*_2_/*K*_1_, one would expect the doubly bound complex AA·B_2_ to become more probable at a given *K*_1_[AA]_*T*_. This is indeed observed in Fig. 4(c) for *K*_2_/*K*_1_ = 10^7^. There, once [AA]_*T*_ > [B]_*T*_, the AA ligands bind every available B molecule, and overwhelmingly so in doubly-bound AA·B_2_ complexes. This means that there are half as many AA·B_2_ complexes as B molecules, which explains the plateau value 0.5 in Fig. 4(c). Yet, even for large *K*_2_/*K*_1_, *saturating* by AA will again drive [AA·B_2_] down, for the same above-given reason. If, in a practical application, the objective is to bind as many B as possible (for instance to prevent a virus from attaching to a cell surface, see Fig. 2 of ref. 8), using a divalent ligand with *K*_2_/*K*_1_ ≫ 1 may be successful. Beyond [AA]_*T*_ ≈ [B]_*T*_, there is no point in further increasing [AA]_*T*_, as all B will be bound from thereon.

An analytical expression [eqn (E5)] for bell-shaped [AA·B_2_]/[B]_*T*_ curves was found in ref. 27 under the assumption that [B]_*T*_ ≪ [AA]_*T*_. From their expression followed that [AA·B_2_]/[B]_*T*_ reaches a maximal value 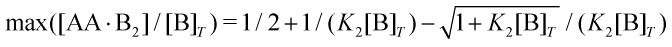 at *K*_1_[AA]_*T*_ = 1/2 and that [AA·B_2_]/[B]_*T*_ should be symmetric around this maximum when plotted against log(*K*_1_[AA]_*T*_).^27^ For *K*_2_/*K*_1_ = 10 and *K*_2_[B]_*T*_ = [0.1,1,10] as used in Fig. 4(b), we find max([AA·B_2_]/[B]_*T*_) = [0.0857,0.268,0.410], which are close to the peak values observed there. For *K*_1_[B]_*T*_ = 0.1, we see that eqn (E5) (grey lines) actually closely follows the numerical data for all *K*_1_[AA]_*T*_ considered. Conversely, for *K*_1_[B]_*T*_ = [1,10], the bell shape of [AA·B_2_]/[B]_*T*_ becomes skewed and shifts away from *K*_1_[AA]_*T*_ = 1/2 to larger *K*_1_[AA]_*T*_. For these *K*_1_[B]_*T*_ values, the assumption [B]_*T*_ ≪ [AA]_*T*_ is incorrect for small *K*_1_[AA]_*T*_. For *K*_1_[AA]_*T*_ = 1 and *K*_1_[B]_*T*_ = 10, for example, one has that [B]_*T*_/[AA]_*T*_ = 10, so the assumption [B]_*T*_ ≪ [AA]_*T*_ underlying eqn (E5) is not justified. For these *K*_1_[AA]_*T*_ and *K*_1_[B]_*T*_ values, it makes more sense to compare the numerical data to eqn (D2), which was derived under the opposite assumption [B]_*T*_ ≫ [AA]_*T*_. Indeed, in the regime of small *K*_1_[AA]_*T*_, the numerical data in Fig. 4(b) is accurately described by eqn (D2) (black dashed lines). Hence, as the ratio [B]_*T*_/[AA]_*T*_ varies during a titration experiment, the analytical expressions for [AA·B]/[B]_*T*_ and [AA·B_2_]/[B]_*T*_ for different [B]_*T*_/[AA]_*T*_ limits hold in different *K*_1_[AA]_*T*_-regimes. Similar observations can be made in Fig. 4(a) and (c). For instance, we see that [AA·B]/[B]_*T*_ is decently described by eqn (E4) for *K*_1_[B]_*T*_ = 0.1 and all considered *K*_1_[AA]_*T*_. Conversely, for *K*_1_[B]_*T*_ = [1,10], we see that [AA·B]/[B]_*T*_ is described by eqn (D1) for small *K*_1_[AA]_*T*_ and by eqn (E4) for large *K*_1_[AA]_*T*_. Similar to [AA·B_2_]/[AA]_*T*_, also [AA·B]/[AA]_*T*_ shifts towards larger *K*_1_[AA]_*T*_ for larger *K*_1_[B]_*T*_.

### 3.3 Adding B to a solution of AA

**Fig. 5:**
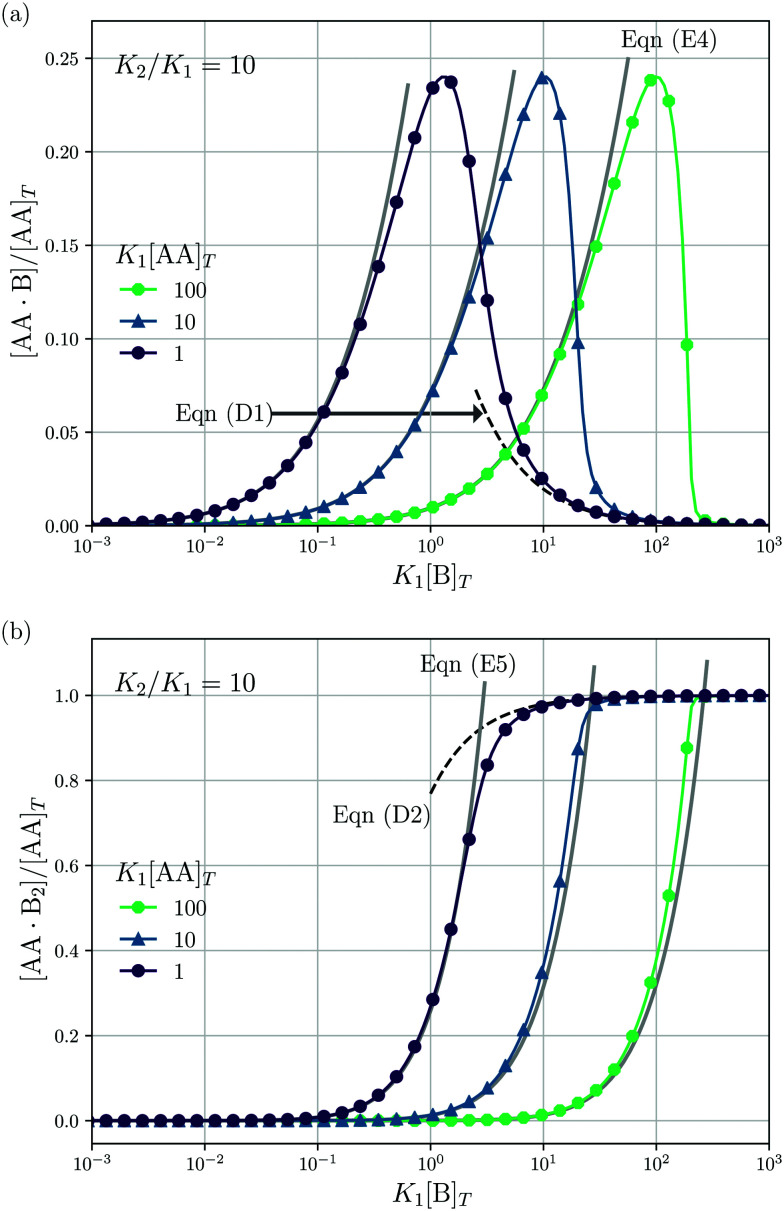
Theoretical predictions for a titration-like experiment wherein [B]_*T*_ increases at fixed *K*_1_, *K*_2_, and [AA]_*T*_. We show [AA·B]/[AA]_*T*_ (a) and [AA·B_2_]/[AA]_*T*_ (b) as a function of *K*_1_[B]_*T*_ for *K*_2_/*K*_1_ = 10 and several *K*_1_[AA]_*T*_. Panel (a) also shows eqn (E4) (grey solid lines) and eqn (D1) (black dashed line); panel (b) also shows eqn (E5) (grey solid lines) and eqn (D2) (black dashed line).

Lastly, we mimic a titration experiment wherein [B]_*T*_ is varied at fixed [AA]_*T*_, *K*_1_, and *K*_2_. As our governing eqn (2) and (3) are not invariant under swapping AA and B—unlike, for example, A and B in the reaction A + B ⇌ AB, see ^ref. 3^—we can expect results different from the previous subsection, wherein [AA]_*T*_ was varied at fixed [B]_*T*_, *K*_1_, and *K*_2_. Fig. 5 shows [AA·B]/[AA]_*T*_ (a) and [AA·B_2_]/[AA]_*T*_ (b) as a function of *K*_1_[B]_*T*_ for *K*_2_/*K*_1_ = 10 and various *K*_1_[AA]_*T*_. Compared to adding AA to a solution of B discussed before, we see that qualitative features of [AA·B] and [AA·B_2_]—sigmoidal and bell shapes—have interchanged. Intuitively, in a solution saturated with B molecules, AA ligands are likely to find two binding partners, yielding high [AA·B_2_]. Both [AA·B]/[AA]_*T*_ and [AA·B_2_]/[AA]_*T*_ shift towards larger *K*_1_[B]_*T*_ for larger *K*_1_[AA]_*T*_. As in Fig. 4, in Fig. 5 we also show predictions from eqn (D1) and (D2) (black dashed lines) and eqn (E4) and (E5) (grey solid lines). These analytical expressions are again seen to agree fairly well with the numerical results for [AA·B]/[AA]_*T*_ and [AA·B_2_]/[AA]_*T*_, in this case either for large or small *K*_1_[B]_*T*_. Unlike Fig. 4, where eqn (D1) and (D2) depend on *K*_1_[B]_*T*_, eqn (D1) and (D2) in Fig. 5 are independent of *K*_1_[AA]_*T*_.

## 4 Conclusion

We have described the reversible binding of two identical monovalent molecules B to a divalent ligand AA. The same process has been studied before, but only in concentration limits of either many more divalent ligands than monovalent molecules or *vice versa*. We considered any ratio of concentrations of divalent ligands and monovalent molecules instead. Our theoretical work is rooted in the classical reaction-rate equations for the above reaction. At steady state, these reduce to four coupled equations for the concentrations [AA], [B], [AA·B], and [AA·B_2_] of unbound, partly bound, and fully bound molecule-ligand complexes, with dependence on the four parameters *K*_1_, *K*_2_, [AA]_*T*_, and [B]_*T*_. We have highlighted the role played by the different parameters by mimicking three different experiments wherein we either varied [AA]_*T*_ and [B]_*T*_ at fixed [B]_*T*_/[AA]_*T*_, varied [AA]_*T*_ at fixed [B]_*T*_, or varied [B]_*T*_ at fixed [AA]_*T*_. In these different scenarios, the concentrations [AA·B] and [AA·B_2_] exhibit a rich and nontrivial dependence on *K*_1_[B]_*T*_ (or *K*_1_[AA]_*T*_), *K*_2_/*K*_1_, and [B]_*T*_/[AA]_*T*_. Specifically, curves for [AA·B] and [AA·B_2_] as a function of *K*_1_[AA]_*T*_ or *K*_1_[B]_*T*_ are either sigmoidal or (roughly) bell shaped. Which of these two shapes appears depends on the varied parameters and on the values of the fixed parameters. In one case [Fig. 2(a)], we observed a transition from a sigmoidal to a bell shape with increasing [B]_*T*_/[AA]_*T*_. Only in the limits [B]_*T*_/[AA]_*T*_ → ∞ and [B]_*T*_/[AA]_*T*_ → 0 do we recover the results of [Hunter and Anderson, Angewandte Chemie International Edition, 2009, **48**, 7488] and of [Perelson and DeLisi, Mathematical Biosciences, 1980, **48**, 71]; at finite [B]_*T*_/[AA]_*T*_, their results contain errors of 𝒪([AA]_*T*_/[B]_*T*_) and 𝒪([B]_*T*_^3^/[AA]_*T*_^3^), respectively.

Comparable concentrations of reacting species can occur both in *in vivo* and in synthetic biological systems. The constraint of particle conservation in homodivalent ligand-monovalent molecule binding—described in this article—can be especially relevant in cellular contexts, where few molecules of either species may be present. However, for tiny systems with small numbers of particles, the reaction rate equation-type modelling that underlies our results breaks down. Our results could then be used as a benchmark in more accurate stochastic models for the same reaction^35^ or in models that account for molecular crowding.^36^ Our work can also be a stepping stone to study how different protein-to-ligand ratios affect heterodivalent interactions^15,37–39^ and the competition between monovalent and divalent receptors for divalent ligands.^31,40^

## Conflicts of interest

There are no conflicts to declare.

## Supplementary Material

## Appendices

### A Derivation of eqn (3) from reaction-rate equations

We repeat eqn (1)

A1

where now *k*_1_ and *k*_2_ and *k*_−1_ and *k*_−2_ are forward and backward reaction rates, respectively. From the law of mass action follow the reaction-rate equations,

A2a

A2b

A2c

A2d

which need to be supplement with initial concentrations of the four species, which we choose as

A3a [AA](*t* = 0) ≡ [AA]_*T*_,

A3b [B](*t* = 0) ≡ [B]_*T*_,

A3c [AA·B](*t* = 0) = 0,

A3d [AA·B_2_](*t* = 0) = 0.

Time-dependent concentrations were studied in ref. 14. Here, we study the steady state, for which eqn (A2a) and (A2b) are identical and eqn (A2c) is the sum of eqn (A2a) and (A2d). Writing *K*_1_ = *k*_1_/*k*_−1_ and *K*_2_ = *k*_2_/*k*_−2_, we arrive at eqn (3) of the main text.

### B General solution to eqn (6)

From hereon, we use the following dimensionless parameters: dimensionless concentrations *x*_1_ = [AA]/[AA]_*T*_, *x*_2_ = [B]/[AA]_*T*_, *x*_3_ = [AA·B]/[AA]_*T*_ and *x*_4_ = [AA·B_2_]/[AA]_*T*_; dimensionless association constants (or, equivalently, “normalized concentration” scales^20^) *κ*_1_ = *K*_1_[B]_*T*_ and *κ*_2_ = *K*_2_[B]_*T*_; the ligand-to-molecule ratio *ξ* = [B]_*T*_/[AA]_*T*_, and the “cooperativity parameter” *α* = *K*_2_/*K*_1_. Using these definitions, we rewrite eqn (2) and (3) to

B1a 1 = *x*_1_ + *x*_3_ + *x*_4_,

B1b *ξ* = *x*_2_ + *x*_3_ + 2*x*_4_,

B1c *x*_3_ = 2*κ*_1_*ξ*^−1^*x*_2_*x*_1_,

B1d

Eqn (4) to

B2a *x*_3_ = 2*κ*_1_*ξ*^−1^(*ξ* − *x*_3_ − 2*x*_4_)(1 − *x*_3_ − *x*_4_),

B2b

Eqn (5) to

B3

and eqn (6) to*ax*_3_^3^ + *bx*_3_^2^ + *cx*_3_ + *d* = 0,*a* ≡ *κ*_1_*κ*_2_ − *κ*_2_^2^,*b* ≡ 2*ξ*(*κ*_1_ − *κ*_2_) − 2*κ*_1_*κ*_2_,*c* ≡ 2*ξκ*_1_(*κ*_2_ − 1) − *ξ*^2^(2*κ*_1_ + *κ*_1_*κ*_2_+ 1),

B4 *d* ≡ 2*ξ*^2^*κ*_1_.

Substituting *x*_3_ = *u* − *a*/3 into eqn (B4) yields

B5

whose solution, with Viète's formula, reads

B6

Depending on the values of *κ*_1_,*κ*_2_, and *ξ*, the determinant *Δ* = −(4*p*^3^ + 27*q*^2^) can be both positive and negative. Hence, for different parameter settings, eqn (6) has either three real roots or one real and two complex roots.

### C No cooperativity, *α* = *K*_2_/*K*_1_ = 1

In absence of cooperativity (*K*_1_ = *K*_2_) we have that *κ*_1_ = *κ*_2_ ≡ *κ* and eqn (B4) simplifies to

C1 −2*κ*^2^*x*_3_^2^ + *ξ*[2*κ*(*κ* − 1) − *ξ*(*κ* + 1)^2^]*x*_3_ + 2*κξ*^2^ = 0.

The positive solution to the quadratic eqn (C1) reads

C2a

with

C2b

For the interpretation of the plateaus at *κ* ≫ 1 in Fig. 3(a), we note that, for *κ* ≫ 1 and *ξ* ≈ 1,

C3 *x*_3_ = *ξ*(1 − *ξ*/2) + 𝒪(*κ*^−1^).

Eqn (C3) breaks down for *ξ* > 2, as *x*_3_ < 0 corresponds to [AA·B] < 0, which is nonphysical.

Inserting eqn (C2) into *x*_4_ [eqn (B3)] and *θ* ≡ *x*_3_/2 + *x*_4_ yields

C4a

C4b

We note that the case of *K*_2_/*K*_1_ = 1 considered here is related to the simpler reaction A + B ⇌ A·B. Comparing two solutions with equal concentrations of [AA] and [A], the number of ligating units in the former solution is twice as high. Replacing *ξ* → 2*ξ* in eqn (C4b) accordingly, this expression reduces to eqn (4.38) of ref. 3 for the scaled concentration [A·B]/[A]_*T*_ at a ligand-to-molecule ratio *ξ* = [B]_*T*_/[A]_*T*_.

In terms of the dimensionless parameters, the Hill coefficient of eqn (8) can be expressed as

C5

where  is such that ; hence,

C6

Inserting eqn (C4b), we find

C7

and

C8

We find that . For smaller *ξ*, the condition of half occupancy in the definition of the Hill coefficient is never fulfilled, leaving *n*_H_ undefined for *ξ* ≤ 1.

### D Few divalent ligands [AA]_*T*_ ≪ [B]_*T*_ (*ξ* ≫ 1)

For *ξ* ≫ 1, eqn (B4) reduces to

D1

Inserting eqn (D1) into eqn (B3) and again taking *ξ* ≫ 1, we find

D2

The occupancy *θ* is found as

D3

Eqn (D1)–(D3) coincide with eqn (S20)–(S22) of ref. 20, which were used to draw Fig. 4 therein.

For the case *ξ* ≫ 1, we find *n*_H_ in terms of the cooperativity parameter *α* by inserting eqn (D3) into eqn (C6),

D4

### E Many divalent ligands [AA]_*T*_ ≫ [B]_*T*_ (*ξ* ≪ 1)

Next, we seek approximate solutions to eqn (B4) for *ξ* ≪ 1. We insert the power series  into eqn (B4), collect terms of equal order in *ξ*, and demand the coefficient of each successive order in *ξ* to be zero. For *n* = 3, we find

E1

We insert eqn (E1) into eqn (B3) and find

E2

Ref. 27 attacked the same problem differently. They stated that [AA] = [AA]_*T*_ holds approximately if [AA]_*T*_ ≫ [B]_*T*_. Then, the term (1 − *x*_3_ − *x*_4_) in eqn (B2a), which stems from [AA] should be replaced by 1, yielding

E3 *x*_3_ = 2*κ*_1_*ξ*^−1^(*ξ* − *x*_3_ − 2*x*_4_),

instead. Inserting *x*_4_ [eqn (B3)] as before now yields

E4

equivalent to eqn (19) of ref. 27.

We insert eqn (E4) into eqn (B3) and find

E5

equivalent to eqn (20) of ref. 27.

As eqn (E4) and (E5) were derived setting *x*_1_ = 1, argued on the basis of *ξ* ≪ 1, the *ξ*-range of validity of these expression is not obvious. Expanding eqn (E4) for small *ξ*,

E6

we see that eqn (E1) and (E6) differ at 𝒪(*ξ*^3^). Practically, setting *κ*_1_ = *κ*_2_ = 10^2^, the two approximations eqn (E1) and (E6) differ from the numerically found root by 0.0001% and 0.52% at *ξ* = 0.1 and 0.5% and 50% at *ξ* = 1, respectively. As expected: for small *ξ*, both approximations are decent and for *ξ* = 1, eqn (E1) performs better.

Likewise, expanding eqn (E5) for small *ξ* yields

E7

Again, differences between eqn (E2) and (E7) appear at 𝒪(*ξ*^3^). Concluding, eqn (19) and (20) of ref. 27, contain errors of 𝒪(*ξ*^3^).

### F Cubic equation for *x*_2_

In Section 2 and Appendix B, we reduced the original four coupled equations in eqn (2) and (3) to a single cubic equation for [AA·B] (or *x*_3_). This was a convenient choice for us as we focused our discussion on [AA·B] and [AA·B_2_]. Yet, as we show next, we may have also derived a cubic equation for [B] instead. From eqn (B1a) and (B1b) we find

F1a *x*_1_ = 1 − *x*_3_ − *x*_4_,

F1b *x*_3_ = *ξ* − *x*_2_ − 2*x*_4_.

From eqn (F1a) and (B1d) we find

F2

F3

Inserting eqn (F1a) and (F1b) into eqn (B1c) we find

F4 *ξ* − *x*_2_ − 2*x*_4_ = 2*κ*_1_*ξ*^−1^*x*_2_(1 − *x*_3_ − *x*_4_) = 2*κ*_1_*ξ*^−1^*x*_2_(1 − *ξ* + *x*_2_ + *x*_4_).

Inserting eqn (F3) gives

F5

which yields

F6 0 = *x*_2_^3^*κ*_1_*κ*_2_ + *x*_2_^2^(2*κ*_1_*κ*_2_+ 2*κ*_1_*ξ* − *κ*_2_*κ*_1_*ξ*) + *x*_2_(*ξ*^2^ + 2*κ*_1_*ξ* − 2*κ*_1_*ξ*^2^) − *ξ*^3^,

or, in our original notation,

F7 0 = [B]^3^*K*_1_*K*_2_ + [B]^2^(2*K*_1_ − *K*_2_*K*_1_[B]_0_ + 2*K*_1_*K*_2_[AA]_*T*_) + [B](1 + 2*K*_1_[AA]_*T*_ − 2*K*_1_[B]_*T*_) − [B]_*T*_.

Eqn (F7) is equivalent to eqn (25) of ref. 29—up to factor 2 discrepancies in a few places, which we trace back to her eqn (15), the counterpart of our eqn (B1c) and (B1d), which does not include prefactors 2 and 1/2. Redefining our *K*_1_ → *K*_1_/2 and *K*_2_ → 2*K*_2_ lifts these discrepancies. Moreover, eqn (F7) is equivalent to eqn (S) of ref. 13 in the case that their “nonreactive fraction parameter” *nr* is set to *nr* = 0.
